# The number of primitive endoderm cells in the inner cell mass is regulated by platelet-derived growth factor signaling in porcine preimplantation embryos

**DOI:** 10.5713/ab.22.0481

**Published:** 2023-02-27

**Authors:** Jong-Nam Oh, Mingyun Lee, Gyung Cheol Choe, Dong-Kyung Lee, Kwang-Hwan Choi, Seung-Hun Kim, Jinsol Jeong, Chang-Kyu Lee

**Affiliations:** 1 Department of Agricultural Biotechnology, Animal Biotechnology Major, and Research Institute of Agriculture and Life Sciences, Seoul National University, Seoul 08826, Korea; 2 Designed Animal and Transplantation Research Institute (DATRI), Institute of Green Bio Science and Technology, Seoul National University, Pyeongchang 25354, Korea

**Keywords:** Epiblast, Pig, Platelet-derived Growth Factor, Preimplantation Embryo, Primitive Endoderm

## Abstract

**Objective:**

Discovering the mechanism of cell specification is important to manipulate cellular lineages. To obtain lineage-specific cell lines, the target lineage needs to be promoted, and counterpart lineages should be suppressed. Embryos in the early blastocyst stage possess two different cell populations, the inner cell mass (ICM) and trophectoderm. Then, cells in the ICM segregate into epiblasts (Epi) and primitive endoderm (PrE). PrE cells in embryos show specific expression of platelet-derived growth factor (PDGF) and its receptor, PDGF receptor A (PDGFRA). In this study, we suppressed PDGF signaling using two methods (CRISPR/Cas9 injection and inhibitor treatment) to provide insight into the segregation of embryonic lineages.

**Methods:**

CRISPR/Cas9 RNAs were injected into parthenogenetically activated and in vitro fertilized embryos. The PDGF receptor inhibitor AG1296 was treated at 0, 5, 10, and 20 μM concentration. The developmental competence of the embryos and the number of cells expressing marker proteins (SOX2 for ICM and SOX17 for PrE) were measured after the treatments. The expression levels of the marker genes with the inhibitor were examined during embryo development.

**Results:**

Microinjection targeting the PDGF receptor (PDGFR) A reduced the number of SOX17-positive cell populations in a subset of day 7 blastocysts (n = 9/12). However, microinjection accompanied diminution of Epi cells in the blastocyst. The PDGF receptor inhibitor AG1296 (5 μM) suppressed SOX17-positive cells without reducing SOX2-positive cells in both parthenogenetic activated and *in vitro* fertilized embryos. Within the transcriptional target of PDGF signaling, the inhibitor significantly upregulated the *Txnip* gene in embryos.

**Conclusion:**

We identified that PDGF signaling is important to sustain the PrE population in porcine blastocysts. Additionally, treatment with inhibitors was a better method to suppress PrE cells than CRISPR/Cas9 microinjection of anti-PDGF receptor α gene, because microinjection suppressed number of Epi cells. The PDGF receptor might control the number of PrE cells by repressing the proapoptotic gene *Txnip*. Our results can help to isolate Epi-specific cell lines from blastocysts.

## INTRODUCTION

The cells that compose the whole body come from a single fertilized egg [1]. Together with continuous cell divisions, lineage-specific signals arise to establish diverse embryonic cell populations. In the preimplantation period, blastomeres form two cell populations, the inner cell mass (ICM) and trophectoderm (TE). Following, ICM cells segregate into epiblasts (Epi) and primitive endoderm (PrE) [2]. The detailed mechanisms of segregation have yet to be unveiled. To address the questions regarding cell fate decisions, researchers have defined starting lines and branching points of lineages. Except for mammals, zygotes undergo molecular gradient through eggs, which gives them the capacity to lead the axis of embryos [3]. However, mammals do not show any signs of segregation at the time of fertilization, and embryonic cells are equivalent in the early stage [4]. Embryos start to show differences among cells, and segregation begins from the 4-cell stage in mice [5].

Lineage markers have been discovered for each lineage to identify the lineage segregation of embryonic cells [6]. To distinguish lineages in a single embryo, immunocytochemistry (ICC) has been used as a conventional method [7,8]. Thus, ICC has a restriction on the scale of targeting markers. Recently, single-cell-based RNA studies have been reported to overcome this limitation. Expression patterns of transcriptional markers were identified from zygotes to blastocysts using quantitative polymerase chain reaction (qPCR) [9]. Furthermore, the RNA-seq technique revealed the transcriptome of embryonic cells at single-cell resolution. Segregation is characterized by the expression levels of lineage-specific marker genes in mice [10], and cells from three germ layers could be sorted clearly by transcriptional profiles in human embryos [11]. Particularly in pigs, qPCR and RNA-seq were conducted with single embryo cells [12,13]. Marker transcripts were defined in those experiments, with *Dab2* representing TE, *Nanog* for Epi, and *Pdgfra* for PrE. Additionally, our previous report showed that SOX17 and SOX2 proteins in porcine embryos can identify PrE and Epi cells, respectively [14].

In the case of PrE, genes encoding platelet-derived growth factor (*PDGF*) ligands and* PDFG* receptors (*PDGFRs*) were suggested to be specific markers. PDGF-related genes showed restricted expression in PrE in mice, and they were also expressed in human preimplantation embryos [15,16]. PDGF signaling is known to be involved in the proliferation and survival of PrE cells [17]. In mouse studies, knockout of PDGFR reduced PrE cells in embryos [17]. Additionally, the number of PrE cells decreased, and the number of TE and Epi cells was maintained with the PDGF signaling inhibitor Gleevec (also known as imatinib) [15]. Together with the results in mice, effect of PDGF suppression on PrE specification needs to be understood in other mammals. To inhibit PDGF signaling, several inhibitors, including AG1296, have been introduced [18]. AG1296 selectively inhibits the PDGF receptor by blocking the kinase activity of the receptor [19]. PDGF-related studies have not been reported in porcine embryos. Therefore, microinjection of anti-PDGFRA and application of PDGF inhibitors to embryos need to be conducted.

In this study, we presented the results of PDGFRA suppression in porcine preimplantation embryos. First, we analyzed the effects of microinjection of anti-PDGFRA clustered regularly interspaced short palindromic repeats (CRISPR)/CRISPR-associated protein 9 (Cas9) on *in vitro* fertilzied (IVF) embryos. Cas9 mRNA and PDGFRA-sgRNAs were injected into fertilized eggs. Developmental competency was monitored during *in vitro* culture of the embryos. SOX17- and SOX2-positive cells were identified on day 7, and the ratio of marker-positive cells was analyzed. Next, we applied a PDGF inhibitor to parthenogenetic activated (PA) and IVF embryos. The inhibitor was applied after fertilization, and developmental competency was calculated. Likewise, we counted the total cell number of embryos and the number of SOX17- and SOX2-positive cells. We also analyzed the relative proportion of marker-presenting cells in day 7 embryos. Furthermore, the expression levels of *PDGF* target genes were quantified to identify the phenotype. Our results suggest suppression of PDGF signaling can be utilized to control embryonic lineages in porcine embryos.

## MATERIALS AND METHODS

The care and experimental use of pigs were approved by the Institute of Laboratory Animal Resources, Seoul National University (SNU-140328-2). Unless otherwise stated, we obtained all chemicals from Sigma-Aldrich Corp. (St. Louis, MO, USA).

### Production of CRISPR/Cas9 vectors for target sequence verification

We searched for the target site of CRISPR/Cas9 on porcine platelet-derived growth factor receptor alpha (*PDGFRA*, Gene ID: 100627123) using the online tool CHOPCHOP (https://chopchop.cbu.uib.no/) [20]. Four target sequences were selected for two genomic sites on PDGFRA (two target sequences for each genomic site; Table 1). The genomic sites were inserted into the cloning site of the pCAG-EGxxFP vector. Oligos for the target sequences were dimerized by slow cooling from 95°C to 25°C, followed by insertion into the guide RNA sequence of the pX330 vector (oligo sequences in Table 2). All vectors were verified by nucleotide sequencing.

### Culture of porcine fetal fibroblasts and plasmid transfection

Basic cell culture and lipofection were carried out following procedures in our previous report [14]. Briefly, EGxxFP and px330 vectors were introduced into porcine fetal fibroblasts at a 1:1 ratio using Lipofectamine 3000 Reagent (Thermo Fisher Scientific, Waltham, MA, USA). We replaced culture media with fresh modified Dulbecco’s modified eagle’s medium (DMEM) with 10% fetal bovine serum 48 hours after lipofection, followed by culture for 2 days. To identify the efficiency of the target sequences, the enhanced green fluorescent protein signal was measured in each sample.

### *In vitro* production of fertilized embryos

The ovaries of prepubertal gilts were obtained from a local slaughterhouse and transferred to the laboratory within warmed saline. Cumulus-oocyte complexes (COCs) were collected by aspirating 3- to 7-mm follicles of prepubertal gilts with a 10-mL syringe and an 18-gauge needle. COCs with compact multiple layers of cumulus cells and fine cytoplasm were collected from aspirated porcine follicular fluid (pFF) and allowed to mature for 44 hours in tissue culture medium 199 (TCM 199) (Gibco, Grand Island, NY, USA) supplemented with 10% pFF, L-cysteine (0.1 mg/mL), sodium pyruvate (44 ng/mL), epidermal growth factor (10 ng/mL), insulin (1 mg/mL), and kanamycin (75 μg/mL) at 39°C. The COCs were matured with 10 IU/mL gonadotropin hormones, pregnant mare serum gonadotropin (Lee Biosolutions, Maryland Heights, MO, USA) and human chorionic gonadotropin only for the first 22 hours. After maturation, cumulus cells were removed from the oocytes with hyaluronidase. Sperm cells were washed twice with Dulbecco’s phosphate buffered saline (DPBS) supplemented with 0.1% bovine serum albumin (BSA) at 1,400 rpm for 3 minutes. Washed sperm (4×10^4^/mL in final concentration) were coincubated with the matured oocytes in 500 μL of modified tris-buffered medium (mTBM) for 4 hours [21]. mTBM consisted of 113.1 mM sodium chloride, 3 mM potassium chloride, 7.5 mM calcium chloride, 20 mM Trizma base, 11 mM glucose, 5 mM pyruvate, 1 mM caffeine, and 0.8% BSA. After this process, eggs were incubated in 5% CO_2_ and 5% O_2_ at 39°C in 20 μL of porcine zygote medium 3 (PZM3) [22]. The cleavage rate was measured on day 2 after insemination. AG1296 (146535-11-7; Sigma, USA) was dissolved with DMSO and diluted in PZM3.

### Microinjection of Cas9 mRNA and gRNAs into IVF embryos

We used commercial Cas9 mRNA (Thermo, USA) and custom synthesized gRNAs. The microinjection procedure was conducted with a microscope (Eclipse TE2000; Nikon, Tokyo, Japan) and micromanipulator (Narishige, Tokyo, Japan) with holding and injection pipettes. We used Femtotip II (Eppendorf, Hamburg, Germany) as an injection pipette. The concentrations of Cas9 RNA and each gRNA were 20 ng/μL and 10 ng/μL, respectively, following a previous report [23]. RNA was injected into fertilized eggs at 2 hours after IVF.

### Immunocytochemistry of embryos

Embryos were washed twice with DPBS supplemented with 0.1% BSA and fixed with 4% paraformaldehyde in DPBS at room temperature (RT) for 15 minutes. Fixed embryos were permeabilized using 0.2% Tween-20 and 0.2% Triton X-100 in DPBS at RT for 15 minutes, followed by blocking with 10% donkey serum in DPBS at RT for 1 hour. Samples were stained with anti-SOX2 (5 μg/mL) and anti-SOX17 (1 μg/mL) in DPBS containing 10% donkey serum at 4°C overnight. After washing 3 times in washing solution (DPBS with 0.2% Tween-20 and 1% BSA for 10 minutes), embryos were incubated with donkey anti-rabbit Alexa594 or donkey anti-goat Alexa488 (Invitrogen, Waltham, MA, USA; 1:5,000) in DPBS with 10% donkey serum at RT for 1 hour. For double staining, samples were stained again with primary and secondary antibodies. The steps were the same, but primary antibody treatment was conducted at RT for 2 hours. All samples were washed 3 times with washing solution after secondary antibody treatment. Immunostained embryos were mounted on a glass slide with Prolong Gold and 4′, 6-diamidino-2-phenylindole (DAPI) (Invitrogen, USA) and cured for more than 24 hours. We described the list of antibodies in Table 3. The digital imaging system for microscope (DS-L1; Nikon, Japan) was used to obtain fluorescence and bright-field images. We used the ImageJ program for images.

### RNA extraction and quantitative polymerase chain reaction

Embryonic RNA was extracted by an Arcturus PicoPure RNA Isolation Kit (Applied Biosystem, Waltham, MA, USA) following the standard manual. cDNA was synthesized from the total RNA of a single embryo with a High-Capacity RNA-to-cDNA Kit (Applied Biosystems, USA) following the standard protocol. Power SYBR Green PCR Master Mix (Applied Biosystems, USA) was used to run quantitative PCR of cDNA samples following the standard manual of the vender. The levels of the transcripts were normalized to the glyceraldehyde-3-phosphate dehydrogenase (GAPDH) expression level by 2^−ΔΔCT^ method [24]. The list of primers is described in Table 2.

### Statistical analysis

Statistical analysis of the data was performed using GraphPad Prism Software (version 5.01; San Diego, CA, USA). Significant differences among experimental groups were determined by one-way analysis of variance followed by Tukey’s multiple comparison test, and unpaired t test was used for the binomial data. A p-value <0.05 was considered significant. Data are presented as the mean±the standard error. The number of embryos used for each experiment is described in Supplementary Table S1.

## RESULTS

### Targeting of PDGFRA using the CRISPR/Cas9 system

**Figure 1:**
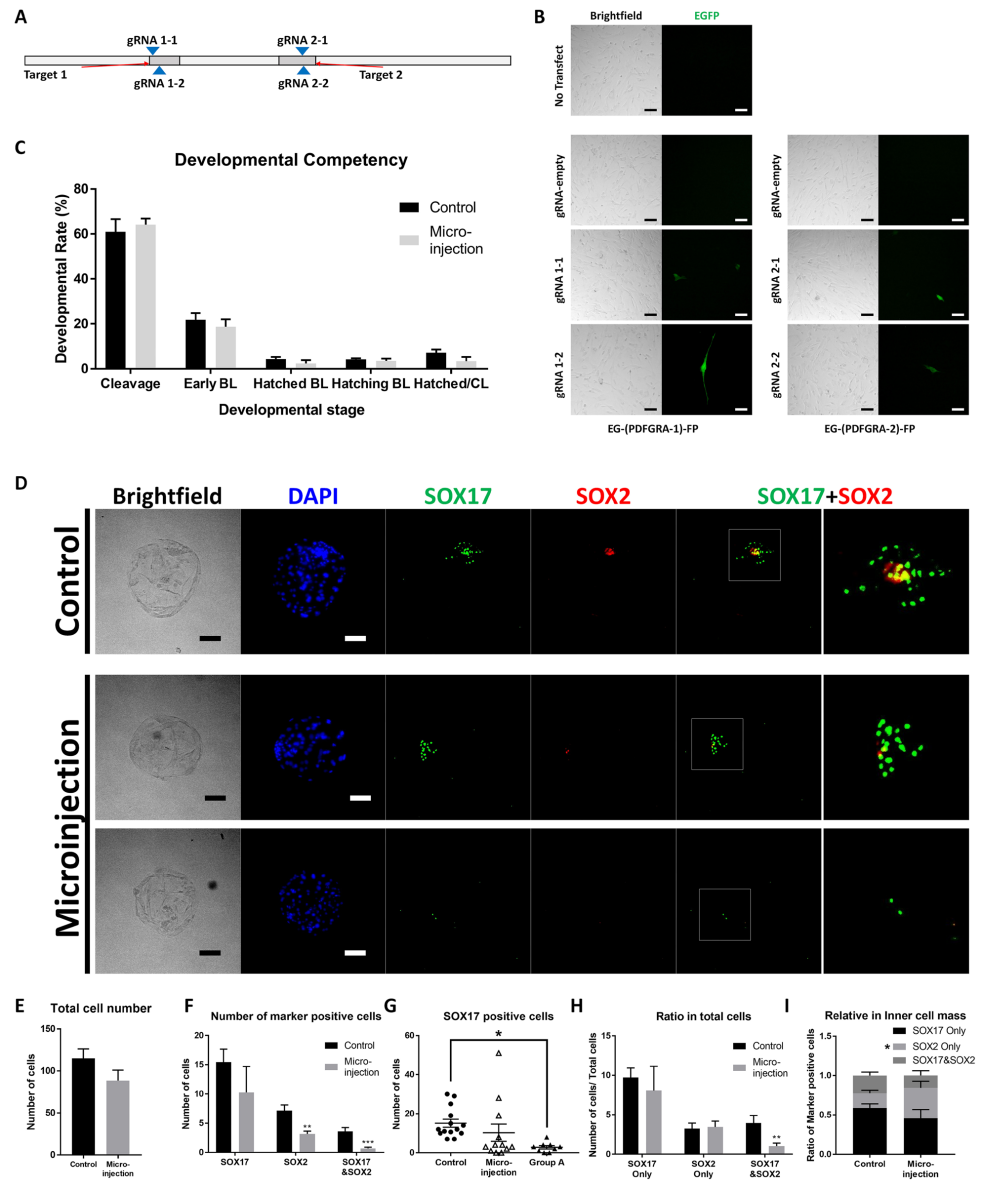
Verification of sgRNA target sequences and the effect of microinjection of anti-PDGFRA CRISPR/Cas9 on IVF embryos. (A) Scheme of target site on the *Pdgfra* gene. Two target sites were selected, and two gRNAs were designed for each target site. (B) Verification of target sequences with porcine fetal fibroblasts. (C) Developmental competency of embryos with or without microinjection. (D) Immunocytochemistry images of day 7 microinjected blastocysts. The rightmost magnified images were originated from merged images for SOX17 and SOX2. (E) Total cell number of day 7 blastocysts with or without microinjection. (F) Number of SOX17- and SOX2-positive cells in day 7 blastocysts. (G) Number of SOX17-positive cells in day 7 blastocysts. Group A is isolated from the microinjected group. (H) Ratio of SOX17- and SOX2-positive cells among total cells in day 7 blastocysts. (I) Ratio of SOX17- and SOX2-positive cells within the inner cell mass in day 7 blastocysts. All scale bars are 100 μm. PDGFRA, platelet-derived growth factor receptor A; IVF, *in vitro* fertilization. * Means significant difference (*p<0.05; ** p<0.01; *** p<0.001).

We designed four gRNA sequences to target two different gDNA sites of PDGFRA (Figure 1A). To evaluate the activity of sgRNAs, modified pCAG-EGxxFP vectors were transfected with px330 vectors containing sgRNA sequences. sgRNAs showed their activity via expression of EGFP (Figure 1B). From the four sequences, we chose sgRNAs #1-2 and #2-1, which are predicted to have higher efficiency and lower off-target effects than #1-1 and #2-2 (Table 1). mRNA of Cas9 and sgRNA #1-2 and #2-1 were injected into IVF embryos. Injection of RNAs showed no significant effect on the developmental competency of embryos (Figure 1C). To evaluate SOX17- and SOX2-positive populations in embryos, ICC and DAPI staining were conducted with day 7 blastocysts (Figure 1D). The total cell number did not show a significant difference between control and RNA-injected embryos (Figure 1E). The numbers of SOX2-positive cells and SOX2-&SOX17-positive cells were reduced, but there was no significant difference in the number of SOX17-positive cells (Figure 1F). In the scatter plot of SOX17-positive cell numbers, 9 of 12 RNA-injected embryos (Group A) showed significantly fewer populations than control embryos (Figure 1G). In view of the ratio of total cells, cells that were positive for both SOX17 and SOX2 were present in lower numbers than the control (Figure 1H). However, when we counted the ratio only within the ICM, only the SOX2 ratio showed a significant difference (Figure 1I).

### Effect of AG1296 on parthenogenetic embryos

**Figure 2:**
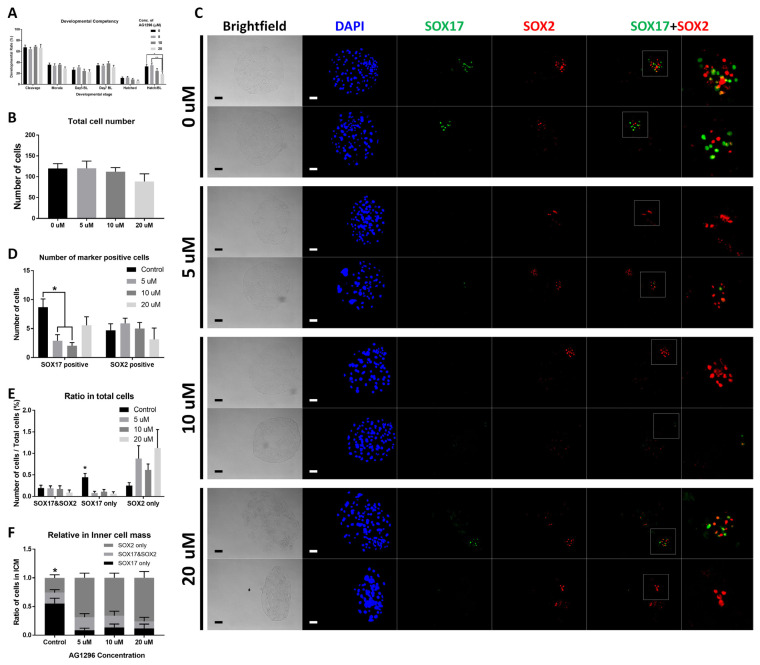
Effect of AG1296 on parthenogenetic activated embryos. (A) Developmental competency of embryos with 0, 5, 10, and 20 μM AG1296. (B) Total cell number of day 7 embryos. (C) Immunocytochemistry images of SOX17 and SOX2 in AG1296-treated embryos. Nuclei were stained with DAPI. The rightmost magnified images were originated from merged images for SOX17 and SOX2. (D) Number of SOX17- and SOX2-positive cells in day 7 blastocysts. (E) Ratio of SOX17- and SOX2-positive cells among total cells in day 7 blastocysts. (F) Ratio of SOX17- and SOX2-positive cells within the inner cell mass in day 7 blastocysts. All scale bars are 100 μm. DAPI, 4′, 6-diamidino-2-phenylindole. * Means significant difference (* p<0.05; ** p<0.01).

AG1296 was applied to parthenogenetic activated (PA) embryos. Treatment with AG1296 did not affect cleavage, morula, blastocyst formation, or hatched blastocyst rate. Within embryos that developed to blastocysts stage, hatching rate was reduced with AG1296 (Figure 2A). At concentrations of 0 to 20 μM, no significant difference was observed in the total number of embryonic cells (Figure 2B). In the ICC, SOX17- and SOX2-positive cell populations were identified (Figure 2C). In the case of cell number, SOX17-positive cells were significantly decreased with 5 and 10 μM AG1296 (Figure 2D). In total cells, the ratio of cells expressing SOX17 was reduced with 5, 10, and 20 μM AG1296 (Figure 2E). Within ICM cells, AG1296 suppressed SOX17-positive cells and number of SOX2-positive cells was increased (Figure 2F).

### Effect of AG1296 on *in vitro* fertilized embryos

**Figure 3:**
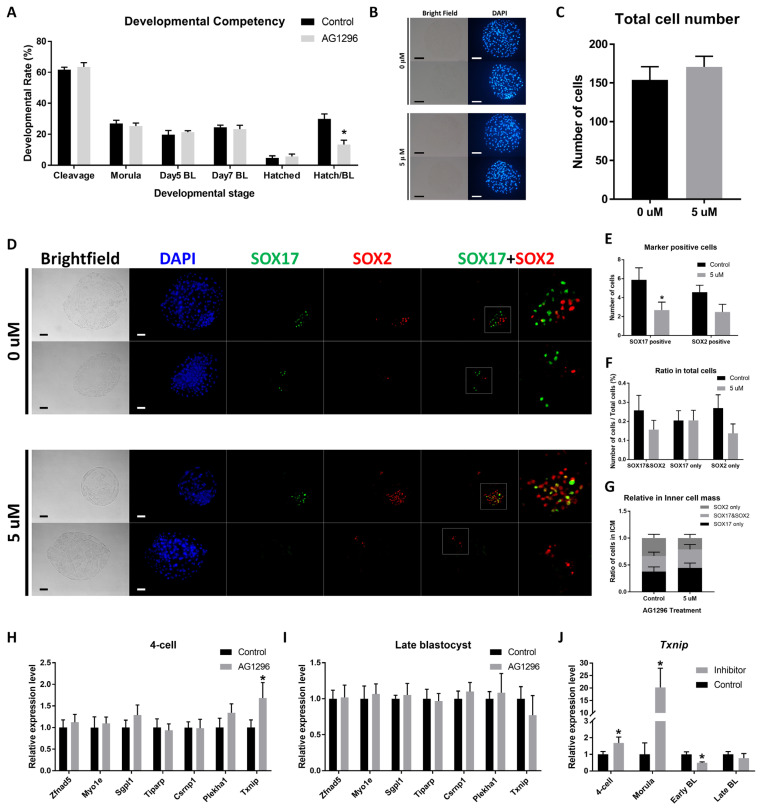
Effect of AG1296 on in vitro fertilized embryos. (A) Developmental competency of embryos with 0 and 5 μM AG1296. (B) Nuclear staining of day 7 blastocysts. (C) Total cell number of day 7 embryos. (D) Immunocytochemistry images of SOX17 and SOX2 in AG1296-treated embryos. The rightmost magnified images were originated from merged images for SOX17 and SOX2. (E) Number of SOX17- and SOX2-positive cells in day 7 blastocysts. (F) Ratio of SOX17- and SOX2-positive cells among total cells in day 7 blastocysts. (G) Ratio of SOX17- and SOX2-positive cells within the inner cell mass in day 7 blastocysts. (H) Relative expression levels of PDGF signaling target genes (*Zfand5*, *Myo1e*, *Sgpl1*, *Tiparp*,* Csrnp1*, *Plekha1*, and* Txnip*) in 4-cell embryos at day 2. (I) Relative expression levels of PDGF signaling target genes (*Zfand5*, *Myo1e*, *Sgpl1*, *Tiparp*, *Csrnp1*,* Plekha1*, and *Txnip*) in late blastocysts at day 7. (J) Relative expression level of *Txnip* in 4-cell, morula, early blastocysts, and late blastocysts. All scale bars are 100 μm. PDGF, platelet-derived growth factor. * Means significant difference (* p<0.05).

We also investigated the effect of AG1296 on IVF embryos. Similar to PA embryos, the hatching rate within blastocysts was decreased with AG1296 (Figure 3A). The number of total cells was not affected by AG1296 treatment (Figure 3B, 3C). In the ICC experiment, AG1296 significantly suppressed the number of SOX17-positive cells (Figure 3D and 3E). However, the ratio of marker-positive cell populations was not significantly different with AG1296 treatment (Figure 3F), and ratio of SOX17 and SOX2-positive cells in ICM did not show any significant difference (Figure 3G). In the qPCR analysis of PDGF downstream genes, *Txnip* was upregulated in 4-cell embryos of the treatment group (Figure 3H), but the other genes (*Zfnad5*, *Myo1e*, *Sgpl1*, *Tiparp*, *Csrnp1*, *Plekha1*) did not show any difference. However, no significant difference was observed in late blastocysts (Figure 3I). Furthermore, the expression level of *Txnip* increased 20-fold in the morula and decreased in early blastocysts treated with AG1296 (Figure 3J).

## DISCUSSION

It is important to understand lineage specification for the control of cell fate and the isolation of lineage-specific cells. Because lineage specification is an autonomous process, triggers should exist at the starting line of segregation to induce embryonic lineages. Many researchers have tried to identify the details of lineage determination in embryos. To establish a specific cell line that represents the embryonic lineage, the counterpart lineage needs to be suppressed. Recent studies in pigs showed that the PDGF receptor is a marker for PrE [12,13]. To discover the potential of the PDGF receptor in porcine embryos, we analyzed the effect of PDGF signaling inhibition in embryos with the lineage markers SOX17 and SOX2. SOX17 and SOX2 are expressed in many cell populations, and they have unique roles in each cell type [25]. In embryonic development, they have specific characteristics to represent embryonic lineages, PrE and Epi. In mice, SOX17 induces the differentiation of embryonic stem cells toward PrE cells [26]. SOX2 is essential for Epi development [27]. Also, SOX17 and SOX2 were also used as protein markers of PrE and Epi, respectively, in pig embryos [28].

After the injection of Cas9 mRNA and sgRNAs, the developmental competency of embryos and cell number of blastocysts were not affected. In the ICC of SOX17, knockout (KO) of PDGFRA was not effective in all samples. However, in the clustered group (Group A in Figure 1G), SOX17-positive cells were reduced significantly. Moreover, two microinjected samples did not possess any SOX17-expressing cells. Therefore, we considered that the KO experiment was partially successful in general and fully successful in some individuals. Additionally, the cell number and ratio of total SOX2- and SOX17-positive cells were decreased in microinjected blastocysts. However, the numbers of SOX2 positive cells and SOX2-only positive cells in the ICM were reduced with the induction of PDGFRA KO. Our purpose in suppressing PDGF signaling was to secure Epi, therefore another method was needed to repress PDGF signaling without a reduction in SOX2-positive cells.

AG1296, a selective and effective inhibitor of the PDGF receptor, was used to treat PA embryos to suppress PDGF signaling [18]. The concentration of AG1296 was determined according to previous reports. A high concentration of AG1296 reduced the hatching rate within blastocysts, but the other components of developmental competency with inhibitor showed no significant difference. AG1296 (20 μM) might exert toxic effects on embryonic development. Additionally, AG1296 did not affect the cell number of day 7 blastocysts. However, the cell number presenting SOX17 was decreased only with 5 μM inhibitor. Moreover, the ratio of SOX17-positive cells in both total cells and ICM decreased. In Epi cells, the cell number and ratio of the SOX2-positive population were not suppressed in the presence of inhibitor. Unlike microinjection, AG1296 could repress SOX17-positive cells with a constant population of SOX2-positive cells. Overall, 5 μM AG1296 showed inhibitory ability without damaging Epi. In further experiments, we used only a 5 μM concentration for treatment.

Inhibitor was applied to IVF embryos in 5 μM concentration. Similar to PA embryos, only the hatching rate in blastocysts was decreased when considering developmental competency, and the total cell number of day 7 blastocysts showed no significant difference. The number of SOX17-positive cells was reduced in the treatment group without a significant difference in SOX2-positive cells. However, the ratio of total cells showed no significant difference. To describe our results in cell populations, we conducted qPCR for the target genes of PDGF signaling with 2-cell embryos and late blastocysts. Among the transcriptional targets of PDGF signaling, only* Txnip* showed a significant difference in 4-cell embryos. The relative expression level of *Txnip* increased 20-fold in the morula with inhibitor, whereas it decreased to half the level in inhibitor-treated early blastocysts. As a downstream target of PDGF signaling, *Txnip* is negatively regulated by activation of the PDGF receptor and induces a proapoptotic role in many cell types [29]. Inhibitors might upregulate *Txnip* and cause PrE cells to die in blastocysts. Therefore, PDGF signaling maintains the PrE cell population by blocking the *Txnip*-mediated apoptotic reaction. Then, a lower level of *Txnip* in inhibitor-treated blastocysts might originate from a low number of PrE cells.

In both microinjection and inhibitor treatment, a reduction in cell number in the SOX17-positive population was observed. This clear phenotype means that PDGF signaling is essential to establish the PrE population in blastocysts. PDGF signaling might sustain the PrE lineage by suppressing the apoptosis pathway. However, microinjection to achieve PDGFRA KO also led to a reduction in Epi cells in blastocysts. To separate the Epi population from a blastocyst, KO may not be an appropriate method in pigs. Nevertheless, treatment with AG1296 suppressed PrE without reducing Epi. In a blastocyst seeding experiment, both Epi and PrE populations were maintained after many passages [30]. Thus, our results could help establish an Epi-specific embryonic cell line.

## CONCLUSION

We verified that PDGF signaling is fundamental for the PrE lineage in porcine blastocysts. Moreover, our results suggested that AG1296 is sufficient to suppress the PrE population and that microinjection of anti-PDGFRA CRISPR/Cas9 inhibits Epi cells. To obtain Epi excluding PrE from blastocysts, the application of a PDGF signaling inhibitor was effective. For further study, a detailed pathway of PDGF signaling through *Txnip* needs to be revealed in embryos. Additionally, cell line establishment from blastocysts using AG1296 will confirm that isolation of the Epi cell population can be extended in subsequent culture after blastocyst seeding. Additionally, other PDGF inhibitors could be challenged to repress the pre-Endo lineage in embryos.

## Figures and Tables

**Table 1:** CHOPCHOP results for gRNA sequences^1)^

Targeting	gRNA number	Target sequence	Genomic location	Strand	Self-complementarity	MM0	MM1	MM2	MM3	Efficiency
Target 1	#1-1	GACCGTTGCAGTCCGATGCTTGG	NC_010450.4:40993720	+	0	0	0	0	0	49.58
	#1-2	TCTGCGTTCCGAACTTACGGTGG	NC_010450.4:40994478	+	1	0	0	0	0	72.66
Target 2	#2-1	ATTGACATGATGGACGACATTGG	NC_010450.4:41018444	+	1	0	0	0	0	64.08
	#2-2	CGACATTGGCATAGACTCCTCGG	NC_010450.4:41018458	+	1	0	0	0	4	60.68

1) Self-complementarity and mismatch were measured based on pig genome, and efficiency was calculated with target sequences.

MM0 = 0 mismatches, MM1 = 1 mismatch, MM2 = 2 mismatches, MM3 = 3 mismatches.

**Table 2:** List of oligo nucleotides. Upper; primers for insertion of gRNA sequences into plasmid vectors, lower; primers for quantitative PCR experiment

Name of oligo		Sequence		
Oligo nucleotides for gRNA insert				
gRNA_#1-1	F	CACCGGACCGTTGCAGTCCGATGCT		
	R	AAACGACCGTTGCAGTCCGATGCTC		
gRNA_#1-2	F	CACCGTCTGCGTTCCGAACTTACGG		
	R	AAACCCGTAAGTTCGGAACGCAGAC		
gRNA_#2-1	F	CACCGATTGACATGATGGACGACAT		
	R	AAACATGTCGTCCATCATGTCAATC		
gRNA_#2-2	F	CACCGCGACATTGGCATAGACTCCT		
	R	AAACAGGAGTCTATGCCAATGTCGC		

**Name of primer**		**Sequence**	**Annealing Tm (°C)**	**Product size (base pairs)**

List of primers used in quantitative PCR				

*Gapdh*	F	TGCTCCTCCCCGTTCGAC	60	100
	R	ATGCGGCCAAATCCGTTC	60	
*Zfand5*	F	AGAGGACAAAATAACTACCCCGAA	60	81
	R	CTGGGCTGAGAAACTGATGGA	60	
*Myo1e*	F	GACGCACAATGCCAACTACC	60	134
	R	ACAGGCTCTTCTGATTTGACCT	60	
*Sgpl1*	F	GTCTCGTGGCAAGAAGGGAA	60	107
	R	AGCGGGTTACTCCATGCAAA	60	
*Tiparp*	F	TCCACACCACCCTCTAGCA	60	130
	R	CCCGAGAGTTGGCTTCTTCA	60	
*Csrnp1*	F	TTCTGTTGCCCCCGAAGTTT	60	82
	R	CATCAAAGGCCACACGACCT	60	
*Plekha1*	F	GACATTGTTGGTGGTGTGCC	60	131
	R	GGCGGTTTCGGAGGAAAGTA	60	
*Txnip*	F	AGGGTTCTGTGAAGGTGATGAG	60	178
	R	GGTTCCTGAGATAATGTGATTGCC	60	

PCR, polymerase chain reaction; *Gapdh*, glyceraldehyde-3-phosphate dehydrogenase; *Zfand5*, zinc finger AN1-type containing 5; *Myo1e*, myosin IE; *Sgpl1*, sphingosine-1-phosphate lyase 1; *Tiparp*, TCDD inducible poly(ADP-ribose) polymerase; *Csrnp1*, cysteine and serine rich nuclear protein 1;* Plekha1*, pleckstrin homology domain containing A1; *Txnip*, thioredoxin interacting protein.

**Table 3:** List of antibodies used in immunocytochemistry experiment

Target	Host	Company	Catalog number	Fluorescent dye	Target/Host	Company	Catalog number
	
Primary antibodies				Secondary antibodies			
SOX2	Rabbit	Millipore	AB5603	Alexa594	Rabbit/Donkey	Invitrogen	A-21207
SOX17	Goat	R&D systems	AF1924	Alexa488	Goat/Donkey	Invitrogen	A-11055

## SUPPLEMENTARY MATERIAL

**Supplementary Table S1.** Number of embryos used in each experiment.
