# Supplementary material for: The number of primitive endoderm cells in the inner cell mass is regulated by platelet-derived growth factor signaling in porcine preimplantation embryos
Source: Anim Biosci. 2023 Feb 27;36(8):1180–9. doi: 10.5713/ab.22.0481 (PMC10330978; doi:10.5713/ab.22.0481)
Supplement: Supplementary file 1 [file ab-22-0481-Supplementary-Table-1.pdf]

Supplementary Table S1. Number of embryos used in each experiment. The same number of embryos were used in the control and experimental groups.

Figure number	Panel						
Fig 1	C	D	F	G	H	I	
# of embryos	7	12	12	12	13	11	
Fig 2	A	B	D	E	F		
# of embryos	8	7	10	7	7		
Fig 3	A	C	E	F	G	H	I	J
# of embryos	4	16	16	16	15	5	5	5
